# Estimated hepatitis C prevalence and key population sizes in San Francisco: A foundation for elimination

**DOI:** 10.1371/journal.pone.0195575

**Published:** 2018-04-11

**Authors:** Shelley N. Facente, Eduard Grebe, Katie Burk, Meghan D. Morris, Edward L. Murphy, Ali Mirzazadeh, Aaron A. Smith, Melissa A. Sanchez, Jennifer L. Evans, Amy Nishimura, Henry F. Raymond, on behalf of End Hep C SF

**Affiliations:** 1 Facente Consulting, Richmond, California, United States of America; 2 University of California, San Francisco, San Francisco, California, United States of America; 3 South African Centre for Epidemiological Modelling and Analysis (SACEMA), Stellenbosch University, Stellenbosch, South Africa; 4 San Francisco Department of Public Health, San Francisco, California, United States of America; 5 Blood Systems Research Institute, San Francisco, California, United States of America; 6 Department of Epidemiology, School of Public Health, Rutgers University, Piscataway, New Jersey, United States of America; Centers for Disease Control and Prevention, UNITED STATES

## Abstract

**Background:**

Initiated in 2016, *End Hep C SF* is a comprehensive initiative to eliminate hepatitis C (HCV) infection in San Francisco. The introduction of direct-acting antivirals to treat and cure HCV provides an opportunity for elimination. To properly measure progress, an estimate of baseline HCV prevalence, and of the number of people in various subpopulations with active HCV infection, is required to target and measure the impact of interventions. Our analysis was designed to incorporate multiple relevant data sources and estimate HCV burden for the San Francisco population as a whole, including specific key populations at higher risk of infection.

**Methods:**

Our estimates are based on triangulation of data found in case registries, medical records, observational studies, and published literature from 2010 through 2017. We examined subpopulations based on sex, age and/or HCV risk group. When multiple sources of data were available for subpopulation estimates, we calculated a weighted average using inverse variance weighting. Credible ranges (CRs) were derived from 95% confidence intervals of population size and prevalence estimates.

**Results:**

We estimate that 21,758 residents of San Francisco are HCV seropositive (CR: 10,274–42,067), representing an overall seroprevalence of 2.5% (CR: 1.2%– 4.9%). Of these, 16,408 are estimated to be viremic (CR: 6,505–37,407), though this estimate includes treated cases; up to 12,257 of these (CR: 2,354–33,256) are people who are untreated and infectious. People who injected drugs in the last year represent 67.9% of viremic HCV infections.

**Conclusions:**

We estimated approximately 7,400 (51%) more HCV seropositive cases than are included in San Francisco’s HCV surveillance case registry. Our estimate provides a useful baseline against which the impact of *End Hep C SF* can be measured.

## Introduction

Despite implementation of hepatitis C virus (HCV) surveillance and inclusion of HCV measures in national U.S.-based surveys, local-level population estimates of HCV burden are lacking. Reliable disease burden estimates are necessary for response planning, and are particularly salient for targeted treatment programs. Highly effective direct-acting antivirals (DAAs) can cure HCV in nearly all people infected,[1] and when paired with prevention programs, offer the first real opportunity to reduce and subsequently eliminate HCV.[2, 3] Multiple strategies for HCV elimination are now being announced at the international,[4] U.S. national,[5] and local levels. [6, 7] As such, HCV prevalence estimation, including within specific high-risk ‘key populations’, is critical for appropriate resource allocation toward elimination.

Historically, efforts to document the HCV burden in San Francisco have included ‘core’ HCV surveillance (mandated reporting of positive HCV antibody or RNA test results), and ‘enhanced’ surveillance, in which approximately 25% of newly confirmed cases are contacted for detailed interviews. These methods, however, do not provide reliable prevalence estimates–a critical measure of disease burden–as the registry excludes people undiagnosed or diagnosed before 2007, and includes those who have died, moved away since testing, or been cured.

San Francisco is on the cusp of a major shift in its approach to HCV. Building upon impressive successes in reducing the city’s HIV burden using interventions tailored through comprehensive annual HIV prevalence estimation, [8] *End Hep C SF* is a new initiative of the San Francisco Department of Public Health, University of California San Francisco (UCSF), and community partners, which coordinates and consolidates resources to achieve HCV elimination. [7] Initiative activities include rapid scale-up of HCV testing and navigation, education and prevention campaigns, and expansion of treatment access through clinical and non-traditional settings. To measure intervention effect, it is necessary to have a valid citywide baseline HCV prevalence estimate, both overall and for key populations. We therefore aimed to estimate the population size and HCV prevalence (proportion seropositive and viremic HCV infections) citywide, including for specific key populations. *End Hep C SF* will apply baseline prevalence estimates to target current prevention and treatment interventions to reduce the number of people with HCV. If San Francisco can significantly reduce the burden of HCV, *End Hep C SF* could provide a model for successful interventions elsewhere.

## Methods

We used a triangulation approach, [9, 10] synthesizing multiple overlapping data sources to produce a reliable baseline estimate of the number of people in San Francisco with anti-HCV antibodies (‘seropositive’) and active HCV infection (‘viremic’). Sources of local HCV-related data include: surveillance case registries, medical records from large private and safety net healthcare providers, blood bank infectious disease testing records, information from San Francisco-based cross-sectional and longitudinal observational studies, and published literature. After a series of discussions with local HCV experts including treating physicians, surveillance staff, and researchers, we decided to limit data to the past 10 years for case registry data, 7 years for published or unpublished study data, and 3 years for medical record treatment data, due to variable reliability and/or relevance of older data from these different datasets. Credible ranges (CRs) for all estimates were derived from 95% confidence intervals (CIs) of population size and prevalence estimates (i.e., the lower bound of the CR is the lower bound of the 95% CI for the population size of a subgroup multiplied by the lower bound of the 95% CI for the prevalence estimate for that subgroup; the upper bound of the CR is the upper bound of the 95% CI for the population size multiplied by the upper bound of the 95% CI for the prevalence estimate).

### Literature review

We searched MEDLINE, Science Citation Index Expanded, and Embase for relevant literature published between January 2010 and January 2017, using the search strategy outlined in S1 Table. These searches resulted in 133, 371, and 139 abstracts found, respectively. These abstracts were then reviewed by hand [SF, EG], discarding those that were studies from other countries, were HIV-specific, or were about microbiology or treatment outcomes not relevant for estimating prevalence. As a result, 59 unique abstracts were retained for further review (S2 Table).

### Stratification of the San Francisco population

The 2015 American Community Survey (ACS) informed the overall and general population size estimates (PSE). [11] We used data from research and surveillance sources to estimate population sizes for key populations at increased risk for HCV: people who inject drugs (PWID), defined as having injected drugs in the last 12 months; men who have sex with men (MSM); and transgender women (TW). [7] Remaining residents were considered ‘general population,’ and included children; adults who are at a low risk of living with HCV; adults who have a past history of drug injection (but have not injected drugs in the past 12 months), are currently or formerly homeless, and/or are currently or formerly incarcerated; and other subgroups without sufficient data to determine HCV prevalence or population size. To address uneven distribution of infection by age and sex we stratified the general population as follows: children 14 and under (both sexes), and males and females separately for ages 15–49, 50–69 (which in 2015 included people born 1945–1965, known as the “birth cohort” of baby boomers), and 70 and older.

### Population size estimates

#### PWID

**Fig 1:**
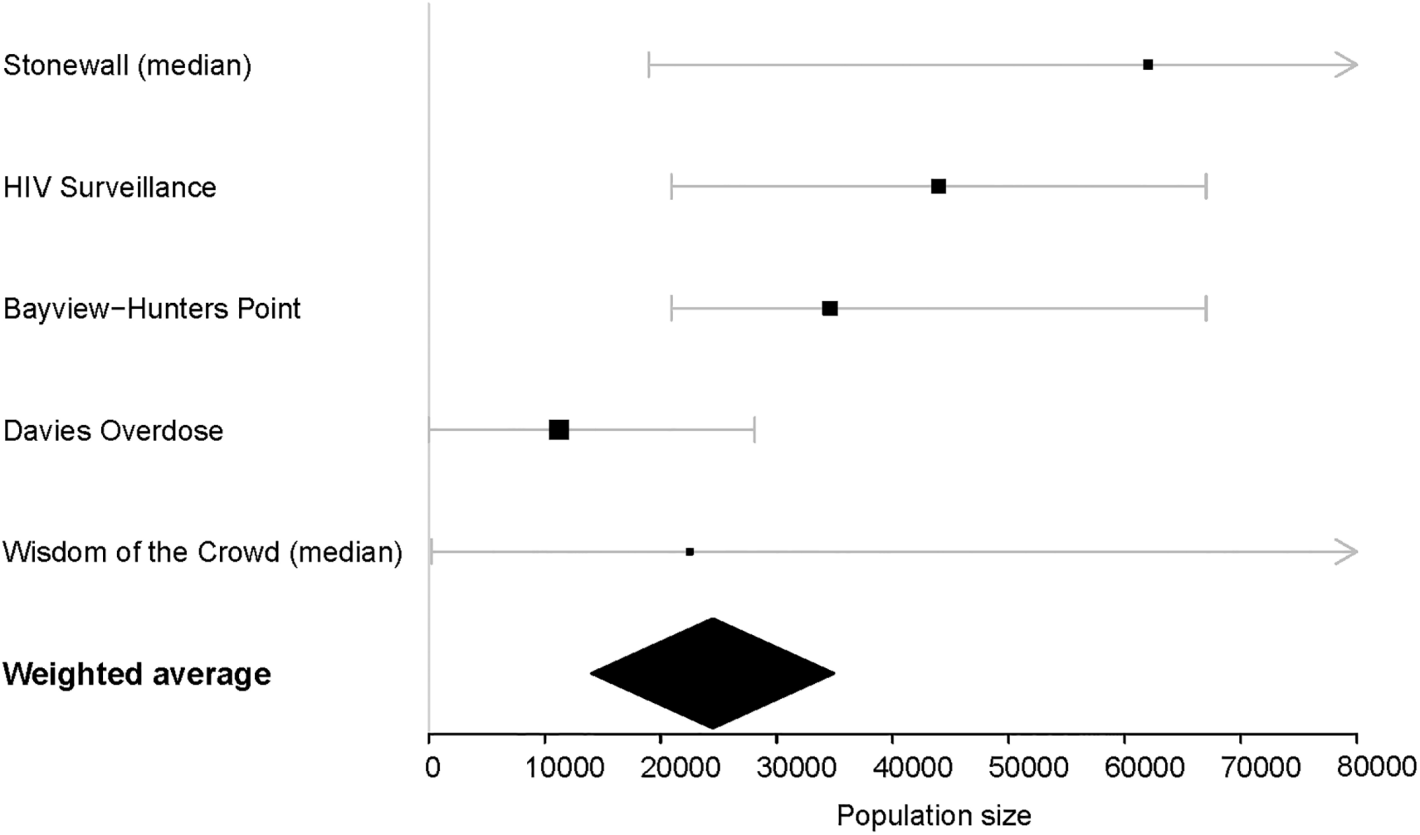
People who inject drugs population size estimates, data inputs [12] and weighted averages*. *Because inverse probability weighting is sensitive to bias in the contributing estimates, from all estimates included in Chen, *et al*. 2016,[12] we excluded: 1) a multiplier estimate based on access of STD testing, which appeared biased by underreporting of injection drug use, and 2) the sequential method, which was strongly influenced by the assumptions of the model, rather than population-based data.

In 2016, Chen *et al*. published a San Francisco PWID PSE for people who have injected drugs in the last 12 months, utilizing the median of a series of multiplier-based estimates, a ‘wisdom of the crowd’ estimate based on NHBS participant responses, and a sequential sampling method based on a number of assumed scenarios. [12] We calculated a weighted average of the individual estimates available in that publication, using inverse variance weighting (see Fig 1). Because this procedure is sensitive to bias in the contributing estimates, we excluded: (1) a multiplier estimate based on access of STD testing, which appeared biased by underreporting of injection drug use, and (2) the sequential method, which was strongly influenced by the assumptions of the model, rather than population-based data.

#### MSM

Two recent papers estimated the San Francisco MSM population size using demographic models to estimate the proportion of adult males in San Francisco who are MSM. [13, 14] We estimated the population by multiplying the median of those proportions (18.5% and 19%) and the number of San Francisco males aged 18 and older in the 2015 ACS. While there is some overlap between the PWID and MSM strata, National HIV Behavioral Surveillance (NHBS) in San Francisco has found quite different HCV prevalence estimates among MSM included in the PWID wave (MSM-PWID) and PWID included in the MSM wave (PWID-MSM). This, along with different patterns in drug use and higher levels of education and employment, suggests MSM-PWID and PWID-MSM are generally distinct groups who are appropriately included in these strata according to their ‘primary characteristic,’ minimizing double-counting in this analysis (Raymond HF, personal communication).

#### TW

San Francisco is currently in its third round (TEACH3) of the Transwomen Empowered to Advance Community Health (TEACH) survey, a cross-sectional HIV behavioral risk survey using Respondent Driven Sampling with TW in San Francisco; TEACH1 was in 2010 and TEACH2 in 2013. Similar to the process used to calculate the PWID PSE, we took a weighted average of seven estimates produced by Wesson and colleagues (2018) via the multiplier method, taking service utilization data from five community clinics, case surveillance, and TEACH1 participants, and using the TEACH2 cohort for the recapture phase. [15] We again excluded Wesson, *et al*’s successive sampling method from this calculation for consistency with our PWID PSE method. The PSE for TW used in this analysis is thought to be specific to TW with ‘low socioeconomic status’; work forthcoming by Raymond, *et al*. demonstrates that only approximately one-third of TW are routinely included in cohort studies or other data sources specific to TW, with the remainder largely invisible and assumed to be part of the general population.

#### General population

The 2015 ACS informed estimates of the number of San Francisco residents not represented in the subgroups outlined above. We produced point estimates and 95% confidence intervals in 5-year age bins by sex, and overall general population strata. These estimates were obtained by deducting key population PSEs from the general population strata according to the participant sex and age distributions in the NHBS 2014 MSM and 2015 PWID San Francisco waves, and the San Francisco TEACH3 cohort. We aggregated the PSEs for the non-key population adult age/sex strata into 3 age bins for each sex. While prevalence estimates for disaggregated strata are more uncertain due to smaller sample sizes, they are programmatically useful, in part because baby boomers (ages 50–69 in 2015) are considered a group with high rates of undiagnosed HCV infection;^12^ further, men and women have generally been found to have different HCV prevalence rates [16] and different patterns of healthcare utilization. [17, 18]

### HCV seroprevalence estimates

#### PWID

**Fig 2:**
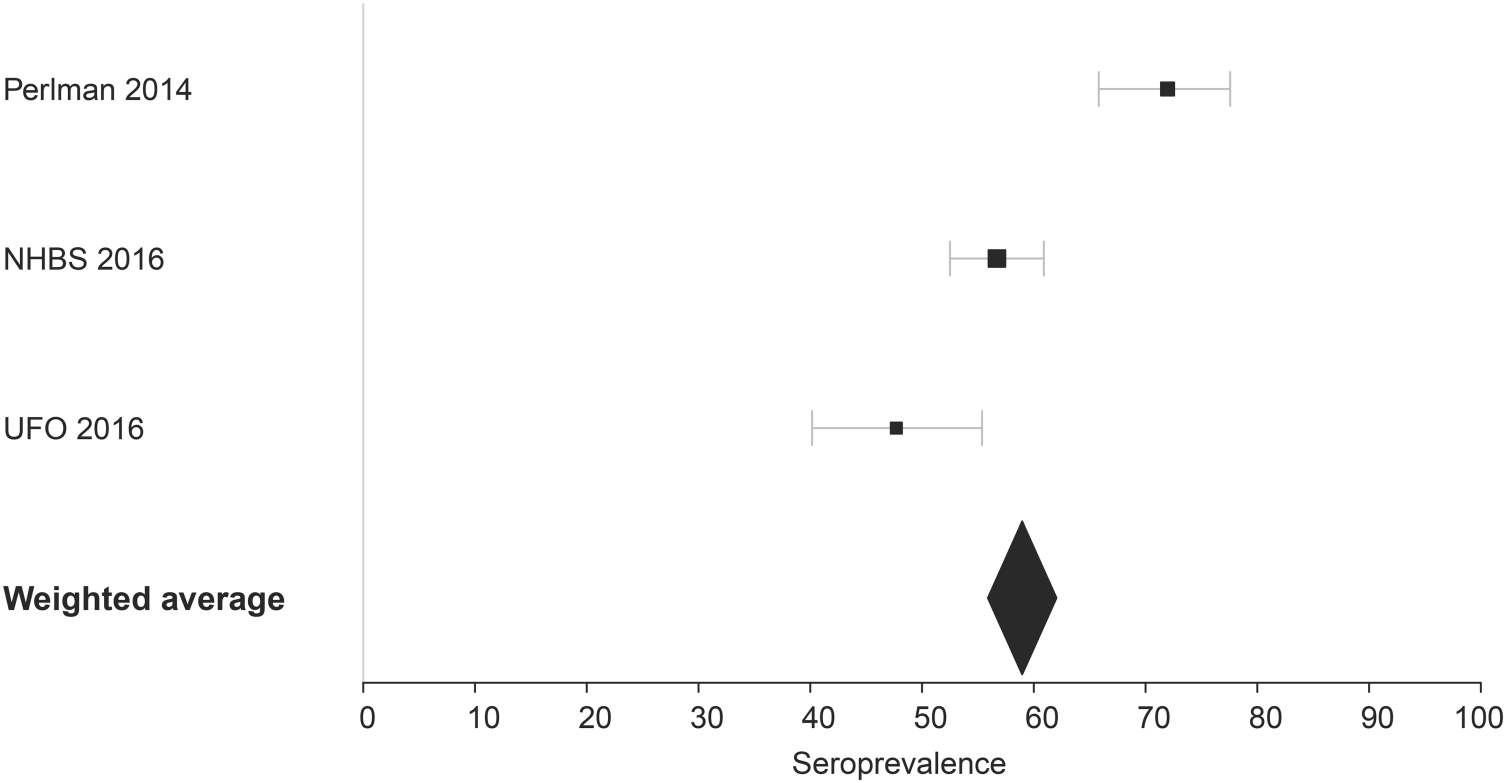
People who inject drugs HCV seroprevalence estimates, data inputs and weighted averages.

We calculated a weighted average of three PWID HCV seroprevalence estimates using inverse variance weighting (fixed effects model); two unpublished estimates were from HCV serological testing in large cohort studies of PWID specific to San Francisco (the 2015 wave of NHBS and the UFO Study from 2015–2016, which enrolls PWID under age 30), and one estimate was from a San Francisco methadone clinic-based study published in 2014 [19] (see Fig 2).

#### MSM

Similar to the method used for PWID, for MSM we used unpublished HCV seroprevalence estimates from serological testing in the 2014 MSM wave of the San Francisco NHBS. We also estimated the HCV burden among HIV-positive MSM in San Francisco, to better inform the targeting of prevention and treatment interventions for co-infected individuals. To do this we used the population size for HIV-positive MSM found in Hughes,[14] and applied the prevalence estimate among HIV-positive MSM from the 2011 NHBS in San Francisco. [20]

#### TW

For ‘low socioeconomic status’ TW, we used unpublished data from serologic testing during the enrollment period of the TEACH3 cohort in San Francisco, which was conducted from May 2016 to February 2017.

#### General population

Data for first-time allogeneic blood donors at the Blood Systems Research Institute (BSRI) from 2006–2015 who were San Francisco residents at the time of donation (n = 40,773) informed the seroprevalence estimate for non-key population adults over 14 years of age. Donors’ ages were normalized to age in 2015 to match the 2015 ACS. To account for potential bias from ‘healthy donor effect’[21] and selection bias related to blood donor eligibility criteria (which exclude PWID, MSM, and others) we applied a weighted adjustment to prevalence. To do this, we compared HCV prevalence in the national REDS-II study of blood donors [22] to the 2012 National Health and Nutrition Examination Survey (NHANES) estimate. [23] We removed respondents who reported ever injecting drugs or men who reported ever having had sex with a man from the NHANES data before estimating prevalence. Population weights were used and clustering was taken into account when calculating prevalence point estimates and standard errors. The ratio between REDS-II chronic infection prevalence and non-key population NHANES chronic infection prevalence was applied as an ‘inflation factor’ (4.9, 95% CI: 2.2–7.7). The delta method was used to incorporate uncertainty in the ‘inflation factor’ into 95% CIs of the resulting prevalence estimates.

#### Children

No known HCV seroprevalence data exist for San Francisco children under age 15. Therefore, we first estimated the number of viremic women of childbearing age (15–49) using a) HCV chronic prevalence estimates from San Francisco first-time blood donations by females of child-bearing age, obtained from BSRI during 2006–2015 (n = 15,506), and the population size for the stratum of non-key population females age 15–49; and b) recent HCV chronic prevalence estimates for PWID from the UFO Study, and the estimated number of female PWID aged 15–49 according to the age and gender distribution of the 2015 wave of NHBS. We then multiplied this number by the birth rate among San Francisco women (n = 1716). This estimated number of births was multiplied by the risk of vertical transmission of HCV for HIV-negative women in the US (0.040, 95% CI: 0.01–0.071) as published in two relevant studies;[24, 25] the number of HIV-infected women in San Francisco is extremely small,[8] and rates of vertical transmission have been shown to be higher among women co-infected with HIV and HCV. [26]

### Calculation of viremic cases

Calculation of the number of people in San Francisco with active HCV viremia has important public health implications. Those who are viremic are targets for secondary prevention interventions, as they can transmit to others, as well as targets for treatment programs. For this reason, we set out to estimate the number of viremic cases, as a subset of the total number of seropositive San Francisco residents.

#### Chronic infections

Point estimates and 95% CIs of prevalence of chronic infection (i.e. active viremia) were available from the UFO Study for PWID, and from BSRI blood donor data for the adult general population. In those strata, the chronic HCV prevalence was directly applied to calculate the estimated number of chronically infected people. For children under 15, in both studies from which the risk of vertical transmission was averaged, successful mother-to-child transmission was defined as the child testing positive for HCV RNA at older than 18 months (when maternal antibodies have waned and spontaneous clearance would typically have already occurred). [26] Since we had no direct estimate of seroprevalence among children or of the proportion of perinatally-infected children who spontaneously clear the infection, the chronic prevalence was used as a conservative estimate of seroprevalence. We found no estimate of chronic prevalence for MSM or TW; therefore, we calculated the weighted average of the proportions of seropositives who were viremic from the NHANES 2012 (weighted analysis), the UFO Study, and BSRI first-time blood donors, and applied that proportion to the estimated numbers of seropositives in the MSM and TW strata.

#### Acute infections

We define ‘acute’ HCV infection as having detectable HCV viremia but still producing a non-reactive result on an anti-HCV antibody test, a window period estimated to last a mean of 60 days from infection, with a range of 20–150 days. [27] For PWID, the acute infection prevalence was taken from the UFO Study. For MSM, we used HCV incidence estimates for MSM reported by Yaphe *et al*,[28] assuming constant (time-invariant) incidence and a mean acute window of 60 days. Window period ranges were used to estimate the credible range of MSM acute prevalence. For TW, no data were found for acute infection prevalence or incidence of HCV infection. Therefore, TW acute prevalence is approximated as the seroprevalence ratio of TW to PWID. In this case, we manually set the acute prevalence credible range bounds to the point estimate for MSM acute prevalence and PWID acute prevalence, respectively, reflecting the general belief that acute prevalence for TW is higher than MSM but lower than PWID in San Francisco. For children, the acute prevalence estimate was set to zero, assuming negligible incidence during the first 14 years of life (excluding vertical transmission). For the remainder of the general population, acute prevalence point estimates and 95% CIs were obtained from the BSRI first-time blood donor data.

#### Treated cases

The total number of patients treated with DAAs in San Francisco was estimated through medical record data of total number of patients treated from 2014–2016, including from the San Francisco Veterans Affairs Medical Center, the San Francisco Health Network (all public health department safety-net clinics and the county hospital), HealthRIGHT360, the UCSF Medical System, California Pacific Medical Center, various DAA clinical trials, and one methadone clinic with high HCV treatment numbers. Importantly, no treatment data was obtained from three major private providers In San Francisco (Kaiser Permanente San Francisco, Dignity Health, or One Medical); therefore we may overcount the untreated viremic cases. Treatment prior to DAAs was disregarded.

#### Viremic cases

The number of viremic cases was calculated as the total number of chronic cases plus acute cases in each of the strata. The number of known treated cases were not available for each stratum, therefore while cases of known treatment were subtracted from the total, successfully treated and cured individuals were still counted as viremic for each subpopulation.

## Results

Overall, the estimated total number of HCV seropositives was 21,758 (credible range [CR]: 10,274–42,067), with a citywide seroprevalence of 2.5% (CR: 1.2%– 4.9%) (Table 1). Of those, 8,305 (CR: 3,858–15,462) are baby boomers, which represents an estimated seroprevalence of 4.1%. In terms of gender, 15,645 (CR: 7,771–27,861) are men, and 5,803 (CR: 2,370–13,917) are women (see Table 2), representing an estimated seroprevalence of 3.6% and 1.4%, respectively. Of all seropositives in San Francisco, 16,408 are estimated to be viremic cases (CR: 6,505–37,407), with up to 11,922 viremic cases still untreated as of the end of 2016 (CR: 2,019–32,921). Table 3 displays sensitivity analyses.

**Table 1:** Estimated population size, seroprevalence and number Anti-HCV antibody and HCV RNA positive, San Francisco, 2017.

Subpopulation		Population size estimate (PSE)		Seroprevalence (anti-HCV)		# HCV seropositive		Prevalence of acute infection		Prevalence of chronic infection		# Viremic* (acute + chronic)	
		*pt*. *est*.	*(credible range*, *CR)*	*pt*. *est*.	*(CR)*	*pt*. *est*.	*(CR)*	*pt*. *est*.	*(CR)*	*pt*. *est*.	*(CR)*	*pt*. *est*.	*(CR)*
PWID		**24,492**	(14,037–34,946)	**59.0%**	(55.9–62.0)	**14,441**	(7,846–21,676)	**3.4%**	(1.3–7.3)	**42.1%**	(33.8–50.1)	**11,147**	(4,926–20,046)
MSM		**69,466**	(68,069–70,863)	**4.4%**	(2.4–6.4)	**3,057**	(1,634–4,535)	**.08%**	(.02–0.2)	**3.2%**	(1.6–5.0)	**2,264**	(1,099–3,717)
TW (low SES)		**951**	(889–1,013)	**22.1%**	(14.9–29.5)	**211**	(132–299)	**1.2%**	(.08–3.4)	**16.0%**	(9.9–23.1)	**163**	(89–268)
Children		**99,391**	(95,756–103,026)	**0.1%**	(0.0–0.2)	**69**	(7–242)	**0.0%**	(0.0–0.0)	**0.1%**	(0.0–0.2)	**69**	(7–242)
General Population	Men 15–49	**185,452**	(180,070–190,834)	**0.4%**	(0.1–1.1)	**777**	(176–2,135)	**0.0%**	(0.0 –.02)	**0.3%**	(0.0–0.8)	**478**	(88–1,560)
	Men 50–69**	**83,174**	(78,915–87,432)	**1.4%**	(0.3–3.8)	**1,173**	(252–3,361)	**0.0%**	(0.0 –.08)	**1.1%**	(0.2–3.1)	**880**	(167–2,816)
	Men 70+	**33,943**	(31,044–36,843)	**0.6%**	(0.0–5.3)	**207**	(2–1,952)	**0.0%**	(0.0–0.5)	**0.0%**	(0.0–3.5)	**0**	(0–1,460)
	Women 15–49	**225,137**	(221,173–229,102)	**0.2%**	(0.0–0.7)	**504**	(87–1,650)	**0.0%**	(0.0 –.02)	**0.2%**	(0.0–0.7)	**432**	(68–1,549)
	Women 50–69**	**95,718**	(91,682–99,753)	**0.7%**	(0.1–2.5)	**694**	(107–2,455)	**0.0%**	(0.0 –.09)	**0.4%**	(0.0–1.7)	**347**	(30–1,738)
	Women 70+	**47,321**	(44,268–50,374)	**1.3%**	(0.1–7.5)	**631**	(31–3,775)	**0.0%**	(0.0–0.5)	**1.3%**	(0.1–7.5)	**631**	(31–4,024)
Totals		**865,046**	(825,904–907,188)	**2.5%**	(1.2–4.9)	**21,758**	(10,274–42,067)	**0.1%**	(0.0–0.4)	**1.8%**	(0.7–3.9)	**16,408**	(6,505–37,407)

**PWID**: people who inject drugs; **MSM**: men who have sex with men, a population with a high prevalence of HIV in San Francisco; **TW**: transgender women

* Estimated number HCV viremic include those who have been treated and cured of HCV since becoming chronically infected

** age groups highlight the birth cohort of ‘baby boomers,’ which includes people born 1945–1965 (ages 50–69 in 2015), who are at higher risk for undiagnosed HCV infection. [33]

**Table 2:** Summary of estimated HCV burden by subpopulation. This table demonstrates the percentage of total infections borne by each subpopulation; this helps to illustrate HCV health disparities among subpopulations. For example, 66.4% of all HCV seropositives in San Francisco are PWID, though only an estimated 2.8% of San Francisco residents overall are PWID.

Subpopulation	# HCV seropositive		HCV seroprevalence		% of SF population	% of citywide HCV seropositives	% of citywide HCV viremics*
	*pt*. *est*.	*(credible range)*	*pt*. *est*.	*(credible range)*	*pt*. *est*.	*pt*. *est*.	*pt*. *est*.
PWID	**14,441**	(7,846–21,676)	**59.0%**	(55.9–62.0)	**2.8%**	**66.4%**	**67.9%**
MSM	**3,057**	(1,634–4,535)	**4.4%**	(2.4–6.4)	**8.0%**	**14.0%**	**13.8%**
TW (low SES)	**211**	(132–299)	**22.1%**	(14.9–29.5)	**0.1%**	**1.0%**	**1.0%**
Baby Boomers	**8,305**	(3,858–15,462)	**4.4%**	(2.2–7.5)	**23.5%**	**38.2%**	**37.6%**
Men	**15,745**	(7,771–27,861)	**3.8%**	(2.0–6.5)	**50.9%**	**72.4%**	**71.7%**
Women	**5,803**	(2,370–13,917)	**1.5%**	(0.6–3.4)	**49.0%**	**26.7%**	**27.3%**

* As treatment data were not available by subpopulation, estimated number HCV viremic include those who have been treated and cured of HCV since becoming chronically infected.

**Table 3:** Sensitivity analysis of key point estimates used in final calculations. This table highlights a series of point estimates used to calculate the results in Table 1, along with two variations for estimate, demonstrating the impact that different assumptions (see “description”) would have had on the final calculations (see “Total # viremics”).

**Estimate**		**Description**	**PSE**	**# Viremic PWID**	**Total # viremics**
**PWID PSE**	**Current point estimate**	Weighted average of selected individual estimates used in the Chen^13^ analysis, as described in Methods	24492	11,147	**16,408**
	**Variation 1**	PWID PSE used in Chen^13^ paper (median of individual estimates included in that analysis)	22500	10,241	**15,511**
	**Variation 2**	Weighted average of all individual estimates used in the Chen^13^ analysis	9711	4,420	**9,752**
**Estimate**		**Description**	**seroprevalence**	**# Viremic PWID**	**Total # viremics**
**PWID seroprevalence**	**Current point estimate**	Weighted average of NHBS, UFO study, and Perlman^19^ seroprevalence estimates for San Francisco (fixed effects model, untransformed)	0.590	11,147	**16,408**
	**Variation 1**	Weighted average of NHBS, UFO study, and Perlman^19^ seroprevalence estimates for San Francisco (fixed effects model, logit-transformed)	0.579	10,958	**16,218**
	**Variation 2**	Weighted average of NHBS, UFO study, and Perlman^19^ seroprevalence estimates for San Francisco (random effects model, logit-transformed)	0.581	11,004	**16,263**
	**Variation 3**	Weighted average of NHBS and UFO study seroprevalence only (excluding Perlman, which is methadone-focused, fixed effects model, untransformed)	0.537	10,218	**15,475**
	**Variation 4**	Seroprevalence from 2015 wave of NHBS in San Francisco (only comprehensive PWID community survey)	0.567	10,750	**16,009**
**Estimate**		**Description**	**PSE**	**# Viremic MSM**	**Total # viremics**
**MSM PSE**	**Current point estimate**	Median of the MSM proportions estimated by Grey^14^ and Hughes^15^ for San Francisco (applied to 2015 ACS males)	69,466	2,264	**16,408**
	**Variation 1**	Grey^14^ MSM proportion (18.5%) minus 10%, applied to 2015 ACS males (assuming lower actual MSM proportion)	61,686	2,010	**16,043**
	**Variation 2**	Hughes^15^ MSM proportion (19%) plus 10%, applied to 2015 ACS males (assuming higher actual MSM proportion)	77,431	2,524	**16,489**
**Estimate**		**Description**	**inflation factor**	**# Viremic gen pop**	**Total # viremics**
**General population (ages 15+) seroprevalence**	**Current point estimate**	Ratio between REDS-II chronic infection prevalence and non-key population NHANES chronic infection prevalence	4.9	2,768	**16,408**
	**Variation 1**	BSRI seroprevalence estimates used for sex and age bins (no inflation factor)	0	558	**14,044**
	**Variation 2**	Doubling of the inflation factor used in the current estimate, to more heavily adjust for ‘healthy donor effect’	10	5,581	**19,090**

For PWID, the PSE was 24,492 people (CR: 14,037–34,946) with an HCV seroprevalence of 59.0% (95% CI: 55.9%– 62.0%); thus an estimated total of 14,441 HCV antibody-positive PWID (CR: 7,846–21,676), with an estimated 11,147 viremic infections (CR: 4,926–20,046). For MSM, the PSE was 69,466 people (CR: 68,069–70,863) with an HCV seroprevalence of 4.4% (CR: 2.4%– 6.4%), with 73% of infections being among HIV-positive MSM; thus an estimated total of 3,057 HCV seropositive MSM (CR: 1,634–4,535), with an estimated 2,264 viremic infections (CR: 1,099–3,717). Of those MSM with HCV viremia, 1,656 (CR: 823–2,748) are estimated to be co-infected with HCV and HIV. For ‘lower socioeconomic status’ TW, the PSE was 951 (CR: 889–1,013), with an HCV seroprevalence of 22.1% (CR: 14.9%– 29.5%); thus an estimated total of 211 HCV seropositive TW (CR: 132–299), and 163 viremic infections (CR: 89–268). For children ages 14 and under, the estimated total HCV seropositive and viremic cases was 69 (CR: 7–242). Applying the ‘inflation factor’ to the BSRI prevalence estimates for each of the general population strata resulted in a total estimate of 3,986 non-key population San Francisco residents over age 14 who are HCV seropositive (CR: 655–15,329).

Beyond the estimates of total HCV infections without each subpopulation, the distribution of infection in San Francisco is also important. People who have injected drugs in the last 12 months comprise 67.9% of viremic HCV cases in the city, but only an estimated 2.8% of the total population of San Francisco. MSM comprise 13.8% of viremic HCV cases, and make up 8.0% of the total population. ‘Lower socioeconomic status’ TW comprise 1.0% of viremic cases, ten times their total population percentage of 0.1%. Baby boomers (including those who are PWID, MSM, and TW) comprise 37.6% of viremic cases, and only 23.5% of the total San Francisco population. Men (regardless of age or risk group) comprise 71.7% of viremic cases despite being roughly half of the San Francisco population. More detail illustrating HCV-related health disparities in San Francisco is available in Table 2.

## Discussion

Our estimate of 21,758 seropositive individuals and 2.5% seroprevalence is higher than the national NHANES seroprevalence estimate of 1.4% (95% CI: 0.9%– 2.0%). Compared to the U.S. overall, San Francisco has a greater proportion of people within key populations, leading to a higher overall seroprevalence. Our findings suggest approximately 7,400 (51%) more HCV seropositives than are in the San Francisco Department of Public Health Viral Hepatitis Surveillance Registry, a de-duplicated database of all people testing HCV positive in San Francisco (antibody or RNA) since 2007. [29] Given that many people still counted in the registry may have moved out of San Francisco or died, this suggests more people in San Francisco have been screened and diagnosed than nationally, where only half of the 3.5 million chronically HCV-infected Americans are estimated to have been diagnosed. [30]

There are noteworthy disparities in San Francisco’s HCV burden, with PWID experiencing a disproportionate proportion of disease. Despite PWID making up only 2.8% of the population, 67.9% of viremic HCV infections are among PWID. MSM and TW, who together make up an estimated 8.1% of the population, account for 14.8% of viremic infections. Though men comprised 50.8% of the San Francisco population in 2015, 71.7% of estimated viremic infections are among men. These disparities have important implications when addressing the approximately 7,400 people with undiagnosed HCV and the roughly 12,000 people currently viremic and presumed untreated. Maximally effective interventions will be closely targeted to subpopulations, including ensuring appropriate service locations. Resources must be allocated for PWID, MSM and TW community programs to provide HCV testing and navigation to care.

Our analysis is subject to a few limitations. First, due to limited community-driven data sources for HCV among MSM, TW, and the general population, the precision of estimates for HCV seroprevalence, acute prevalence, and chronic proportion may be reduced. Nonetheless, our study provides the first population size and HCV prevalence estimate for TW in San Francisco. Second, our triangulation of multiple data sources required numerous assumptions and extrapolations that increased estimate uncertainty; therefore we used ‘credible ranges’ to demonstrate outer plausible boundaries instead of attempting to calculate 95% confidence intervals, which imply greater statistical precision. One prominent example of this is our estimate of the prevalence of chronic HCV among children under age 15: our calculation ignored in and outmigration of both children and women during 2000–2015, and assumed that PWID have the same live birth rate as the general population. Similarly, acute prevalence of TW is unknown but estimated through the use of prevalence in other key populations to set plausible bounds. Third, the overall city estimate relied heavily on studies of key populations, which are subject to important geographic and sociodemographic selection biases. Fourth, lack of available information led to the inclusion of numerous high-prevalence subpopulations in the “general population” category, such as people who have a past history of drug injection but have not injected drugs in the past 12 months, people who are currently or formerly homeless, or people who are currently or formerly incarcerated. To address this we applied an ‘inflation factor’ as described in the Methods; however, this approach has limited accuracy. Finally, our source data represent timepoints across 2000–2016, rather than a single point in time (2015). Trends in prevalence over that time period have been ignored due to limited data availability; this could influence the accuracy of the estimates.

These estimates provide a valuable baseline against which the impact of *End Hep C SF* can be measured. Additional refinement using more precise data could improve both the baseline and future estimates. For example, PSE and HCV prevalence data for people with a past history of drug injection, people who are currently or formerly homeless, and people who have a history of incarceration would enhance estimate quality and usability. Additional analyses of this type with stratification by race/ethnicity may also help to illustrate existing disparities in HCV burden. Dynamical HCV transmission models can estimate the intervention effects on disease incidence and prevalence. *End Hep C SF* is working to apply these study findings to projection models to set city-wide HCV elimination targets.

Importantly, local HCV estimates highlight the scope of the epidemic and identify subgroups most needing prevention and treatment support. *End Hep C SF* is a citywide initiative through which many resources are being coordinated and consolidated, with an eye toward HCV elimination. Several conditions make San Francisco an ideal place for a groundbreaking HCV elimination effort, including a history of implementing cutting-edge, evidence-based interventions such as syringe access, opiate agonist therapy, health coverage for uninsured individuals, overdose prevention programs, and early adoption of universal HIV treatment regardless of CD4 cell count. However, *End Hep C SF* also includes a focus on data, improving and aligning research and surveillance of HCV citywide and in key populations, making better use of existing data to guide programs and policies leading to elimination. Findings from this analysis will lead to more explicit targeting of these types of prevention and treatment efforts toward PWID, MSM, TW, and men overall, in addition to baby boomers; future efforts to better understand disparities related to race/ethnicity would also be useful for better targeting strategies.

Untreated HCV has considerable population health impact; HCV is responsible for one-quarter of all cases of hepatocellular carcinoma cirrhosis, and mortality from HCV nationally has now surpassed mortality from HIV, making it the most deadly infectious disease in the U.S. [31] San Francisco has one of the highest rates of liver cancer in the nation,[32] resulting in liver transplants or death. Unchecked HCV transmission has considerable societal cost, including expensive publicly-funded treatment and rising healthcare expenditures due to HCV-related morbidity. While the study applied data sources specific to San Francisco, the methods could be applied by any region with local data sufficient for triangulation.

## Supporting information

S1 Table Literature search strategy. We searched MEDLINE, Science Citation Index Expanded, and Embase for relevant literature published between January 2010 and January 2017, using the search strategy outlined below. These searches resulted in 133, 371, and 139 abstracts found, respectively. These abstracts were then systematically reviewed, discarding those that were studies from other countries, were HIV-specific, or were about microbiology or treatment outcomes not relevant for estimating prevalence. As a result, 59 unique abstracts were retained for further review.
(PDF)

S2 Table Abstracts retained for further review after literature search. Fifty-nine unique abstracts were retained for further review after following the literature search methods described in S1 Table. These abstracts are listed below.
(PDF)

S3 Table Summary of data sources. The table below summarizes the main sources of data for each population size and HCV prevalence estimate contained in this analysis.
(PDF)
