# Correction: Estimated hepatitis C prevalence and key population sizes in San Francisco: A foundation for elimination

**DOI:** 10.1371/journal.pone.0200866

**Published:** 2018-07-12

**Authors:** Shelley N. Facente, Eduard Grebe, Katie Burk, Meghan D. Morris, Edward L. Murphy, Ali Mirzazadeh, Aaron A. Smith, Melissa A. Sanchez, Jennifer L. Evans, Amy Nishimura, Henry F. Raymond, on behalf of End Hep C SF

In the fourth sentence of the Results section, the number of viremic cases is incorrect. The correct sentence is: Of all seropositives in San Francisco, 16,408 are estimated to be viremic cases (CR: 6,505–37,407), with up to 12,257 viremic cases still untreated as of the end of 2016 (CR: 2,354–33,256).
