# Supplementary material for: Estimated hepatitis C prevalence and key population sizes in San Francisco: A foundation for elimination
Source: PLoS One. 2018 Apr 11;13(4):e0195575. doi: 10.1371/journal.pone.0195575 (PMC5895024; doi:10.1371/journal.pone.0195575)
Supplement: S1 Table — We searched MEDLINE, Science Citation Index Expanded, and Embase for relevant literature published between January 2010 and January 2017, using the search strategy outlined below. These searches resulted in 133, 371, and 139 abstracts found, respectively. These abstracts were then systematically reviewed, discarding those that were studies from other countries, were HIV-specific, or were about microbiology or treatment outcomes not relevant for estimating prevalence. As a result, 59 unique abstracts were retained for further review. (PDF) [file pone.0195575.s001.pdf]

Table S1. Literature Search Strategy

We searched MEDLINE, Science Citation Index Expanded, and Embase for relevant literature published between January 2010 and January 2017, using the search strategy outlined below. These searches resulted in 133, 371, and 139 abstracts found, respectively. These abstracts were then systematically reviewed, discarding those that were studies from other countries, were HIV-specific, or were about microbiology or treatment outcomes not relevant for estimating prevalence. As a result, 59 unique abstracts were retained for further review.

Database	Search Strategy	Abstracts Found	Relevant Abstracts Retained*
MEDLINE	#1 Epidemiology[MeSH] OR Incidence[MeSH] OR Prevalence[MeSH] OR Cross-Sectional Studies[MeSH] OR Cohort Studies[MeSH] OR epidemiolog*[tw] OR prevalence[tw] OR incidence[tw] OR cross-section*[tw] OR cohort*[tw]<br>*AND*<br>#2 Hepatitis C[MeSH] OR hepatitis C[tw] OR hep C[tw] OR *HCV*[tw] OR anti-HCV OR HCV-RNA [tw]<br>*AND*<br>#3 San Francisco [tw] OR California [tw] OR Seattle [tw] OR San Diego [tw] OR Los Angeles [tw]	133	22
Science Citation Index Expanded	#1 TS = (Incidence OR Prevalence OR Cross-Sectional OR Cohort OR epidemiolog*)<br>*AND*<br>#2 TS = (Hepatitis C OR hep C OR HCV* OR anti-HCV OR HCV-RNA)<br>*AND*<br>#3 CI = San Francisco OR PS = San Francisco	371	31
Embase	#1 'incidence'/exp/mj OR 'incidence' OR 'prevalence'/exp/mj OR 'prevalence' OR 'cross-sectional study'/exp/mj OR 'cross-sectional study' OR 'cohort analysis'/exp OR 'cohort analysis' OR epidemiolog*<br>*AND*<br>#2 'hepatitis c'/exp/mj OR 'hepatitis c' OR 'hepatitis c antibody'/exp/mj OR 'hepatitis c antibody' OR 'hepatitis c virus genotype 1'/exp OR 'hepatitis c virus genotype 1' OR 'hepatitis c virus genotype 3'/exp/mj OR 'hepatitis c virus genotype 3' OR 'hepatitis c virus genotype 2'/exp OR 'hepatitis c virus genotype 2' OR 'hepatitis c virus subtype 1b'/exp OR 'hepatitis c virus subtype 1b' OR 'hepatitis c virus subtype 1a'/exp OR 'hepatitis c virus subtype 1a' OR 'hepatitis c virus subtype 3a'/exp OR 'hepatitis c virus subtype 3a' OR 'hepatitis c virus subtype 2a'/exp OR 'hepatitis c virus subtype 2a' OR 'hepatitis c virus subtype 2b'/exp OR 'hepatitis c virus subtype 2b' OR 'hcv-rna'<br>*AND*<br>#3 'california'/exp OR 'california':ab,ti OR 'san francisco':ab,ti	139	18

*Duplicate abstracts were retained across databases; a total of 59 unique abstracts were obtained from the totals noted in this column.
