# Supplementary material for: Estimated hepatitis C prevalence and key population sizes in San Francisco: A foundation for elimination
Source: PLoS One. 2018 Apr 11;13(4):e0195575. doi: 10.1371/journal.pone.0195575 (PMC5895024; doi:10.1371/journal.pone.0195575)
Supplement: S2 Table — Fifty-nine unique abstracts were retained for further review after following the literature search methods described in S1 Table. These abstracts are listed below. (PDF) [file pone.0195575.s002.pdf]

Table S2. Abstracts Retained for Further Review After Literature Search

Fifty-nine unique abstracts were retained for further review after following the literature search methods described in Table S1. These abstracts are listed below.

	First Author	Title	Journal	Pub Year
1	Atsma, F.	The healthy donor effect: a matter of selection bias and confounding	Transfusion	2011
2	Backus, L.I.	Hepatitis C Virus Screening and Prevalence Among US Veterans in Department of Veterans Affairs Care	JAMA Internal Medicine	2013
3	Beijer, U.	Prevalence of tuberculosis, hepatitis C virus, and HIV in homeless people: a systematic review and meta-analysis.	Lancet Infect Dis	2012
4	Buffington, J.	Low prevalence of hepatitis C virus antibody in men who have sex with men who do not inject drugs	Public Health Rep	2007
5	CDC	Vital signs: evaluation of hepatitis C virus infection testing and reporting - eight U.S. sites, 2005-2011	MMWR	2013
6	CDC	Surveillance for Viral Hepatitis - United States, 2014	N/A (Report)	2016
7	CA Dept of Public Health	Chronic hepatitis C, San Francisco, 2013	N/A (Report)	2014
8	CA Dept of Public Health	Chronic hepatitis C in California: 2013 Executive Summary	N/A (Report)	2014
9	Chen, Y-H.	Estimated number of people who inject drugs in San Francisco, 2005, 2009, and 2012	AIDS Behavior	2016
10	Chew, K.	Low prevalence of hepatitis C co-infection in recently HIV-infected minority men who have sex with men in Los Angeles: a cross-sectional study	BMC Infectious Diseases	2015
11	Coffin, P.O.	Trends in use of health care and HIV prevention services for persons who inject drugs in San Francisco: results from National HIV Behavioral Surveillance 2005-2012	Drug Alcohol Depend	2015
12	Coffin, P.O.	Clinically-Ascertained and Undiagnosed Hepatitis C Incidence Among Non-Injecting HIV-Positive Men-Who-Have-Sex-with-Men	N/A (Poster)	2012
13	Collier, M.G.	Awareness of HCV infection among persons who inject drugs in San Diego, California	American J Pub Health	2014
14	Cummins, C.A.	Hepatitis C Infection Among Hispanics in California	J of Addictive Diseases	2015
15	Currie, S.L.	A prospective study to examine persistent HCV reinfection in injection drug users who have previously cleared the virus	Drug Alcohol Depend	2008
16	Denniston, M.M.	Chronic Hepatitis C Virus Infection in the United States, National Health and Nutrition Examination Survey 2003 to 2010	Annals of Int Med	2014
17	Dias, P.T.	Temporal changes in HCV genotype distribution in three different high risk populations in San Francisco, California	BMC Infectious Diseases	2011

18	Dodd, R.Y.	Development of a multisystem surveillance database for transfusion-transmitted infections among blood donors in the United States	Transfusion	2016
19	Edlin, B.R.	Toward a More Accurate Estimate of the Prevalence of Heptitis C in the United States	Hepatology	2015
20	Evans, J.L.	Concordance of risk behavior reporting within HCV serodiscordant injecting partnerships of young injection drug users in San Francisco, CA	Drug Alcohol Depend	2014
21	Freiman, J.M.	Current Practices of Screening for Incident Hepatitis C Virus (HCV) Infection Among HIV-Infected, HCV-Uninfected Individuals in Primary Care	Clin Infect Dis	2014
22	Garfein, R.S.	HCV infection prevalence lower than expected among 18-40-year-old injection drug users in San Diego, CA	J Urban Health	2012
23	Gelberg, L.	Prevalence, distribution, and correlates of hepatitis C virus infection among homeless adults in Los Angeles	Public Health Rep	2012
24	Gish, R.G.	Data supporting updating estimates of the prevalence of chronic hepatitis B and C in the United States	Hepatology	2015
25	Guiltinan, A.M.	Increased all-cause, liver, and cardiac mortality among hepatitis C virus-seropositive blood donors	Am J Epidemiology	2008
26	Hennessey, K.A.	Prevalence of infection with hepatitis B and C viruses and co-infection with HIV in three jails: a case for viral hepatitis prevention in jails in the United States	J of Urban Health	2008
27	Hermanstyne, K.A.	The association between use of non-injection drug implements and hepatitis C virus antibody status in homeless and marginally housed persons in San Francisco	J Public Health	2012
28	Kittikraisak, W.	Incarceration among young injectors in San Francisco: Associations with risk for hepatitis C virus infection	J of Substance Use	2006
29	Leyva, Y.	Per-contact infectivity of HCV infection and reinfection in association with receptive needle sharing exposures in a prospective cohort of young injection drug users in San Francisco, CA	N/A (Poster)	2016
30	Lin, O.N.	HCV Prevalence in Asian Americans in California	J Immigr Minor Health	2016
31	Manos, M.M.	Characteristics and clinical management of a large cohort of Hepatitis C patients from a northern California integrated health plan	Hepatology	2012
32	Manos, M.M.	Birth cohort based screening for hepatitis C: The view from a managed care setting	Hepatology	2010
33	Munn, M.S.	Surveillance Findings from Hepatitis C Virus Test and Cure Program: Screening of High Risk Birth Cohort and Descriptive Analysis of Hepatitis C Patient Population		2016
34	Murphy, E.L.	Risk Factors for Hepatitis C Virus Infection in United States Blood Donors	Hepatology	2000
35	Murphy, E.L.	Hepatitis C virus prevalence and clearance among US blood donors, 2006-2007: associations with birth cohort, multiple pregnancies, and body mass index	J Infect Dis	2010

36	Murphy, E.L.	Demographic determinants of hepatitis C virus seroprevalence among blood donors	JAMA	1996
37	Murphy, E.L.	Liver disease, antiretrovial treatment and behavioral risk factors in a retrospective cohort of HCV seropositives detected at the time of blood donation	N/A (Poster)	2009
38	Nguyen, K.	Prevalence and presentation of hepatitis B and C virus (HBV and HCV) infection in Vietnamese Americans via serial community serologic testing	J Immigr Minor Health	2014
39	Nyamathi, A.	Understanding correlates of hepatitis C virus infection among homeless recently paroled men	J Forensic Nurs	2013
40	Page, K.	HCV screening and infection awareness in a cohort of HIV infected and uninfected homeless and marginally housed women in San Francisco, California	BMC Public Health	in press
41	Page, K.	Acute Hepatitis C Virus Infection in Young Adult Injection Drug Users: A Prospective Study of Incident Infection, Resolution, and Reinfection	JID	2009
42	Page, K.	Frequent Longitudinal Sampling of Hepatitis C Virus Infection in Injection Drug Users Reveals Intermittently Detectable Viremia and Reinfection	Clin Infect Dis	2013
43	Page-Shafer, K.	Testing strategy to identify cases of acute hepatitis C virus (HCV) infection and to project HCV incidence rates	J Clin Micro	2008
44	Perlman, D.C.	Viral hepatitis among drug users in methadone maintenance: associated factors, vaccination outcomes, and interventions	J of Addictive Diseases	2014
45	Raymond, H.F.	Hepatitis C infection among men who have sex with men, San Francisco, 2011	Sex Transm Dis	2012
46	Raymond, H.F.	Hepatitis C prevalence among HIV-positive MSM in San Francisco: 2004 and 2008	Sex Transm Dis	2011
47	Riley, E.D.	Antiretroviral therapy, hepatitis C virus, and AIDS mortality among San Francisco's homeless and marginally housed	JAIDS	2005
48	Roth, A.M.	Patterns of drug use, risky behavior, and health status among persons who inject drugs living in San Diego, California: a latent class analysis	Subst Use Misuse	2015
49	Sanchez, M.	Viral hepatitis surveillance: SFDPH	N/A (presentation)	2016
50	Sanchez, M.	Epidemiology of the Viral Hepatitis-HIV Syndemic in San Francisco: A Collaborative Surveillance Approach	Public Health Rep	2014
51	Sarkar, M.	Racial/Ethnic Differences in Spontaneous HCV Clearance in HIV Infected and Uninfected Women	Digestive Diseases and Sciences	2013
52	Smith, D.J.	Spontaneous viral clearance of hepatitis C virus (HCV) infection among people who inject drugs (PWID) and HIV-positive men who have sex with men (HIV+ MSM): a systematic review and meta-analysis	BMC Infectious Diseases	2016

53	Strehlow, A.J.	Hepatitis C among clients of health care for the homeless primary care clinics	J Health Care Poor Underserved	2012
54	Terrault, N.A.	Sexual transmission of hepatitis C virus among monogamous heterosexual couples: the HCV partners study	Hepatology	2013
55	Tracy, D.	Higher risk of incident hepatitis C virus among young women who inject drugs compared with young men in association with sexual relationships: a prospective analysis from the UFO Study cohort	BMJ Open	2014
56	Tseng, F.C.	The inverse relationship between chronic HBV and HCV infections among injection drug users is associated with decades of age and drug use	J Viral Hepatitis	2008
57	Tsui, J.I.	Association of opioid agonist therapy with lower incidence of hepatitis C virus infection in young adult injection drug users.	JAMA Internal Medicine	2014
58	Uccellini, L.	HCV RNA levels in a multiethnic cohort of injection drug users: human genetic, viral and demographic associations	Hepatology	2012
59	Vickerman, P.	The More You Look, the More You Find: Effects of Hepatitis C Virus Testing Interval on Reinfection Incidence and Clearance and Implications for Future Vaccine Study Design	JID	2012
