# Supplementary material for: Estimated hepatitis C prevalence and key population sizes in San Francisco: A foundation for elimination
Source: PLoS One. 2018 Apr 11;13(4):e0195575. doi: 10.1371/journal.pone.0195575 (PMC5895024; doi:10.1371/journal.pone.0195575)
Supplement: S3 Table — The table below summarizes the main sources of data for each population size and HCV prevalence estimate contained in this analysis. (PDF) [file pone.0195575.s003.pdf]

Table S3. Summary of Data Sources

The table below summarizes the main sources of data for each population size and HCV prevalence estimate contained in this analysis.

Subgroup	Estimate	Data source
PWID	PSE	Chen YH, McFarland W, Raymond HF. Estimated Number of People Who Inject Drugs in San Francisco, 2005, 2009, and 2012. AIDS and behavior. 2016;20(12):2914-21.
	HCV prevalence	National HIV Behavioral Surveillance, San Francisco, 2015 (unpublished; available via HFR)
		UFO Study, San Francisco, 2016 (unpublished; available via MDM)
		Perlman DC, Jordan AE, McKnight C, Young C, Delucchi KL, Sorensen JL, et al. Viral hepatitis among drug users in methadone maintenance: associated factors, vaccination outcomes, and interventions. Journal of addictive diseases. 2014;33(4):322-31.
MSM	PSE	Grey JA, Bernstein KT, Sullivan PS, Purcell DW, Chesson HW, Gift TL, et al. Estimating the Population Sizes of Men Who Have Sex With Men in US States and Counties Using Data From the American Community Survey. JMIR Public Health Surveill. 2016;2(1):e14.
		Hughes A, Chen YH, Scheer S, Raymond HF. A novel modeling approach for estimating patterns of migration into and out of San Francisco by HIV status and race among men who have sex with men. Journal of Urban Health. 2017;94(3):350-363.
	HCV prevalence	National HIV Behavioral Surveillance, San Francisco, 2014 (unpublished; available via HFR)
		Raymond HF, Chu P, Nieves-Rivera I, Louie B, McFarland W, Pandori M. Hepatitis C infection among men who have sex with men, San Francisco, 2011. Sexually transmitted diseases. 2012;39(12):985-6.
TW	PSE	Wesson P, Oabazard R, Wilson EC, McFarland W, Raymond HF. Estimating population size of transwomen in San Francisco using multiple methods, 2013. International Journal of Transgenderism. 2017.
	HCV prevalence	TEACH3 cohort, San Francisco, 2017 (unpublished; available via HFR)
General Population	PSE	United States Census Bureau / American Fact Finder. Age and Sex: 2015 American Community Survey 1-Year Estimates. U.S. Census Bureau's American Community Survey Office, 2015.
	HCV prevalence	Data for first-time allogeneic blood donors at the Blood Systems Research Institute (BSRI) from 2006-2015 who were San Francisco residents at the time of donation (unpublished; available via ELM)
		Dodd RY, Notari EP, Nelson D, Foster GA, Krysztof DE, Kaidarova Z, et al. Development of a multisystem surveillance database for transfusion-transmitted infections among blood donors in the United States. Transfusion. 2016.
		National Center for Health Statistics, Centers for Disease Control and Prevention (CDC). National Health and Nutrition Examination Survey Data (NHANES) 2011-2012. Hyattsville, MD2012.
